# Morphoea induced by vaping

**DOI:** 10.1002/ski2.279

**Published:** 2023-09-27

**Authors:** Robert Harrington, Marion Leahy, Jack Roberts, Mary E. Laing

**Affiliations:** ^1^ Department of Dermatology Galway University Hospital Galway Galway Ireland; ^2^ Department of Pathology Galway University Hospital Galway Galway Ireland

## Abstract

Case report of Morphoea induced by the use of electronic cigarettes.

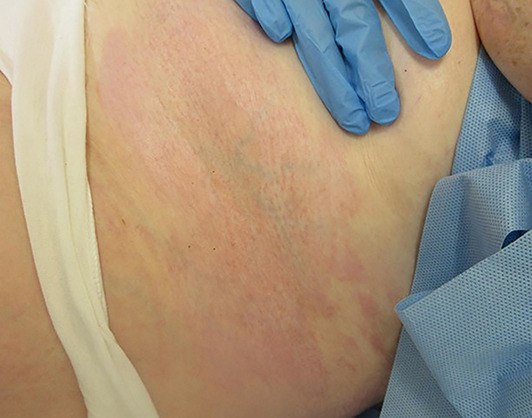

Dear Editor,

Electronic cigarette use or ‘vaping’ has been linked to a number of cutaneous complications including discoid lupus,^1^ contact dermatitis, oral mucosal lesions and thermal injury.^2^ Morphoea secondary to drug and chemical exposures is also well documented in the literature.^3^, ^4^ However, to our knowledge, no case of morphoea induced by the use of electronic cigarettes has previously been reported.

An otherwise well 63‐year‐old female was referred to the dermatology clinic with multiple patches of tight, shiny skin on her left breast and abdomen (Figure 1). The patient reported associated pruritus and some tenderness in the affected areas. She had no personal or family history of relevance and was not on any regular medications. Of note in her social history, the patient was a thirty‐pack year smoker of conventional cigarettes, but in the months prior to the development of symptoms, had switched to electronic cigarettes or “vapes.” Her chosen vape liquid was a nicotine free product and her device contained a wick made of silica. Her laboratory investigations revealed a negative antinuclear antibody and systemic sclerosis profile, normal eosinophils and no hypergammaglobulinaemia. A skin biopsy was performed showing abnormal, swollen dermal collagen bundles as well as a perivascular infiltrate of plasma cells, lymphocytes and histiocytes (Figure 2). These findings were consistent with the clinically suspected diagnosis of morphoea.

**FIGURE 1:**
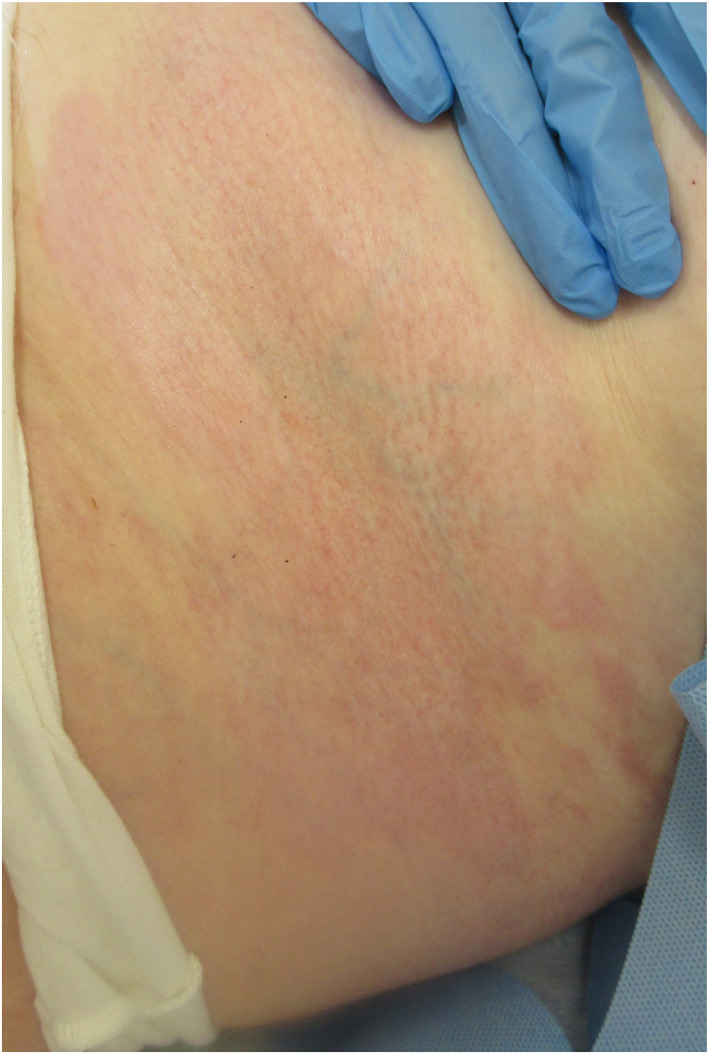
Clinical image of tight, shiny, hyperpigmented skin on the patient's abdomen, clinically consistent with morphoea.

**FIGURE 2:**
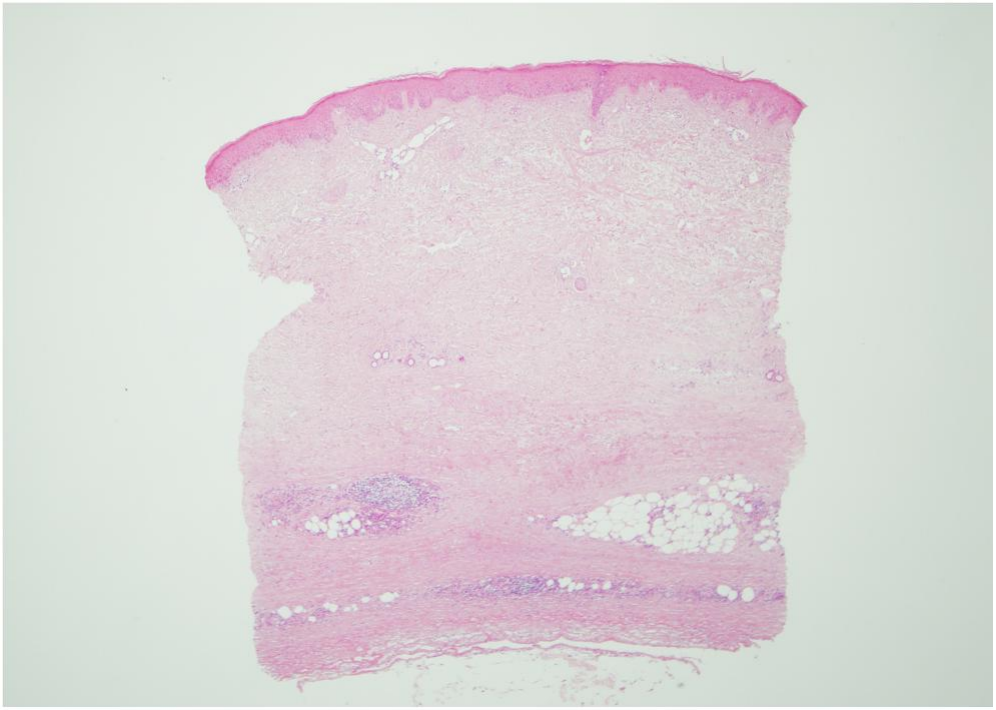
Skin biopsy showing characteristic square shape, abnormal, swollen dermal collagen bundles and a perivascular infiltrate of plasma cells, lymphocytes and histiocytes. The process extends into the subcutaneous fat.

The patient stopped using e‐cigarettes except for very occasional social use. She also switched to a more modern e‐cigarette device which did not utilise silica. Outside of this significant reduction in vaping, the patient opted for topical emollients alone to treat her morphoea. After 6 weeks, her symptoms had almost completely resolved with only mild hyperpigmentation evident on exam. At a further review 12 weeks later her skin had returned completely to normal. She has had no recurrence in her symptoms in the 2 years since her initial presentation. Given the close temporal relationship between commencing vaping and the onset of her symptoms, as well as the rapid resolution of her skin disease after reducing her usage, it was concluded that the patient had suffered chemical‐induced morphoea secondary to vaping.

Electronic cigarette use is on the rise worldwide. There is minimal regulation of these products and little is known currently about their potential adverse effects on health. Thousands of unidentified compounds have been isolated from the liquids and vapour of E‐cigarettes.^5^ Of the identified chemicals, many are known carcinogens and, of relevance to our case some have previously been associated with morphoea and other sclerotic diseases. These include silica, trichloroethylene, formaldehyde and volatile organic compounds such as toluene and benzene.^6^ Interestingly, the wick in our patient's initial device was made of silica, a material which is no longer used in newer devices due to health concerns.

In summary, this patient developed morphoea affecting large areas of her chest and abdomen secondary to vaping. This case highlights a previously unreported potential cutaneous complication of electronic cigarette use.

## CONFLICT OF INTEREST STATEMENT

The authors declare no conflicts of interest.

## AUTHOR CONTRIBUTIONS

**Robert Harrington**: Writing – original draft (lead); Writing – review & editing (lead). **Marion Leahy**: Writing – review & editing (supporting). **Jack Roberts**: Writing – review & editing (supporting). **Mary E. Laing**: Supervision (lead); Writing – original draft (supporting); Writing – review & editing (supporting).

## ETHICS STATEMENT

Not applicable.

## Data Availability

Data sharing not applicable to this article as no datasets were generated or analysed during the current study.
